# Understanding health systems to improve community and facility level newborn care among displaced populations in South Sudan: a mixed methods case study

**DOI:** 10.1186/s12884-018-1953-4

**Published:** 2018-08-10

**Authors:** Samira Sami, Ribka Amsalu, Alexander Dimiti, Debra Jackson, Solomon Kenyi, Janet Meyers, Luke C. Mullany, Elaine Scudder, Barbara Tomczyk, Kate Kerber

**Affiliations:** 1 Department of International Health, Johns Hopkins Bloomberg School of Public Health, 615 N. Wolfe Street, Baltimore, MD 21205 USA; 2 Emergency Health, Save the Children, 2275 Sutter Street, San Francisco, CA 94115 USA; 3 Directorate of Reproductive Health, Ministry of Health, Republic of South Sudan, P.O.Box 336, Juba, South Sudan; 4 UNICEF/University of the Western Cape, 3 UN Plaza, New York, NY 10017 USA; 5 International Medical Corps, Tong ping Area block 3b, Juba, South Sudan; 6 Reproductive Health in Emergencies, Save the Children, 899 North Capitol Street NW, Suite 900, Washington, DC, 20002 USA; 7 Newborn Health, Save the Children, 899 North Capitol Street NW, Suite 900, Washington, DC, 20002 USA; 8 Office of the Director, Center for Global Health, US Centers for Disease Control and Prevention, 1600 Clifton Rd Bldg 21 Rm 9208 MS D-69, Atlanta, GA 30329 USA

**Keywords:** Newborn health, South Sudan, Conflict, Community, Facility, Health system, Displaced populations

## Abstract

**Background:**

Targeted clinical interventions have been associated with a decreased risk of neonatal morbidity and mortality. In conflict-affected countries such as South Sudan, however, implementation of lifesaving interventions face barriers and facilitators that are not well understood. We aimed to describe the factors that influence implementation of a package of facility- and community-based neonatal interventions in four displaced person camps in South Sudan using a health systems framework.

**Methods:**

We used a mixed method case study design to document the implementation of neonatal interventions from June to November 2016 in one hospital, four primary health facilities, and four community health programs operated by International Medical Corps. We collected primary data using focus group discussions among health workers, in-depth interviews among program managers, and observations of health facility readiness. Secondary data were gathered from documents that were associated with the implementation of the intervention during our study period.

**Results:**

Key bottlenecks for implementing interventions in our study sites were leadership and governance for comprehensive neonatal services, health workforce for skilled care, and service delivery for small and sick newborns. Program managers felt national policies failed to promote integration of key newborn interventions in donor funding and clinical training institutions, resulting in deprioritizing newborn health during humanitarian response. Participants confirmed that severe shortage of skilled care at birth was the main bottleneck for implementing quality newborn care. Solutions to this included authorizing the task-shifting of emergency newborn care to mid-level cadre, transitioning facility-based traditional birth attendants to community health workers, and scaling up institutions to upgrade community midwives into professional midwives. Additionally, ongoing supportive supervision, educational materials, and community acceptance of practices enabled community health workers to identify and refer small and sick newborns.

**Conclusions:**

Improving integration of newborn interventions into national policies, training institutions, health referral systems, and humanitarian supply chain can expand emergency care provided to women and their newborns in these contexts.

**Electronic supplementary material:**

The online version of this article (10.1186/s12884-018-1953-4) contains supplementary material, which is available to authorized users.

## Background

Trailing behind progress made in addressing under-five mortality, an estimated 2.6 million newborns die annually in the first 28 days of life [1]. Sub-Saharan Africa has high neonatal mortality rates (NMR, 27.7/1000 livebirths) and stillbirth rates (SBR, 28.7/1000 total births) [1, 2]. The direct causes of death for newborns in many low- and middle-income countries, including conflict-affected settings, are estimated using statistical models, due to a lack of functioning vital registration systems. The three leading causes of neonatal death across the region are preterm birth complications (35%), severe infections (23%) and intrapartum-related conditions (24%) [3]. Countries with the poorest indicators in this region have experienced a humanitarian emergency due to conflict or another form of political instability [2, 4]. South Sudan, facing 5 years of civil war, has high rates of neonatal mortality (37.9 per 1000 live births) and stillbirth (30.1 per 1000 total births) [1, 2].

Substantial evidence exists for the most effective neonatal interventions for reducing mortality across the continuum of care, with interventions during the intrapartum and early postnatal period having the greatest impact [5]. Evidence for the prevention of preterm births in these settings is limited, so identifying and caring for small babies is the best option for addressing this major cause of mortality [6]. Providing essential neonatal interventions in packages, by timing of care and level of service delivery, to address the three main causes of neonatal death can result in better health outcomes, as opposed to a single vertical program [7].

Conflict-affected settings endure long-term public health impact caused by deterioration in the health workforce, facility infrastructure, and medical supply chain [8, 9]. Ongoing insecurity and mass migration further disrupt the health system [10–12]. Additionally, assaults on health workers and facilities have become increasingly common in active conflict [9, 13]. In South Sudan in 2016, where just 43% of health facilities were deemed functional, at least 24 humanitarian aid workers were killed, a health facility in Malakal was burned down in February, just before this study began, and a maternity ward in Juba was shelled during an outbreak of violence in July [14, 15]. Preventing neonatal deaths and stillbirths requires the support of a strong and stable health system that can provide timely care by qualified health workers; subsequently, incidents that damage or weaken the health system can thus increase risks to newborn health and survival.

To address this need, we studied the feasibility of implementing a package of community- and facility-based interventions, as recommended in the *Newborn Health in Humanitarian Settings: Field Guide*, during active conflict in South Sudan. The recently drafted *Field Guide* summarizes existing WHO standards for community- and facility-based newborn care and prioritizes the most critical services for three levels of service delivery during humanitarian crisis [16]. The process for implementing interventions affects outcomes for service delivery (e.g. quality of care) and clinical treatment (e.g. neonatal mortality) [17, 18]. The Consolidated Framework for Implementation Research (CFIR) is useful for understanding what factors moderate implementation [19]. Exploring these constructs within the WHO Health Systems Framework can identify specific entry points to improve the implementation of newborn interventions at critical health system building blocks: leadership and governance, financing, workforce, information systems, essential medical commodities, and service delivery [20]. This framework was also adapted by the *Every Newborn Action Plan* to assess bottlenecks to implementation of critical newborn care interventions and packages [21]*.* In our study, we report findings from a case study exploring factors influencing the implementation of a package of newborn interventions in four displaced person camps during a six-month period. The interventions were implemented in program areas operated by International Medical Corps (IMC), an international humanitarian organization. The specific objectives were to 1) explain the main health system bottlenecks for implementing newborn interventions in displaced person camps, 2) describe facilitators that enabled implementation, and 3) recommend solutions for improving implementation of newborn care.

## Methods

### Design and setting

We used a mixed method case study design to document the implementation of a package of facility- and community-based newborn health interventions by IMC in South Sudan from June to November 2016. Our cases were bound by the context in South Sudan, the second most fragile state with 2.4 million displaced persons in July 2016 [22, 23]. The study was completed in two refugee camps and two internally displaced person (IDP) camps. The refugee camps were in Maban (Gendrassa and Kaya) with a population at the time of the study, primarily from Sudan, of 24,552 and 17,160, respectively [24]. Meanwhile, Malakal IDP camp has a population of 40,448, and Juba IDP camp is home to 27,959 displaced persons [25]. The study included an IMC primary health care center (PHCC) and community health program from each camp, plus a hospital in the Juba IDP camp. Study facilities in Juba operated with skilled birth attendants (SBAs) such as midwives, nurses and clinical officers, while Malakal and Maban employed SBAs alongside traditional birth attendants (TBAs), to maintain services in the evening hours.

### Description of study intervention

With the purpose of improving service delviery and addressing the three main causes of neonatal mortality, the intervention activities consisted of:

1. Clinical training: Each site held local trainings for both facility- and community-based health workers in June 2016. The locally-based medical coordinator and/or midwife supervisor led the trainings, with support from a clinical trainer. Additional trainings were held for health workers who were absent or working during the training period. Sessions included presentations, instructive videos from the Global Health Media Project, and simulations using Laerdal Medical’s NeoNatalie™, PreemieNatalie™ and MamaBreast™ [26–29]. Topics for facility health workers included danger signs during pregnancy, fetal monitoring using the partograph, neonatal resuscitation, immediate newborn care, breastfeeding support, recognition of newborn danger signs, feeding and kangaroo mother care (KMC) for small babies, management of possible severe infections, and postnatal counseling before patient discharge. For community health workers (CHW), topics addressed pregnancy identification and tracking, importance of antenatal care (ANC) and facility-based deliveries, danger signs during pregnancy and for newborns, essential care practices, breastfeeding support, home visits for newborns during the first week of life, and use of a respiratory rate timer, weighing scale and thermometer. Participants included all available community- and facility-based health workers.

2. Supportive supervision: A checklist was designed for supervisors to observe practices learned in the clinical training during a delivery or home visit. Based on the observation, the supervisor provided feedback to the health worker and recorded areas in need of further attention.

3. Distribution of medical commodities: Using the supply list recommended in the *Field Guide*, newborn medical supply kits were procured by the study team from an international medical wholesaler (IMRES) and distributed at the community- and facility- level in each site. The list was modified to avoid waste and delays by adjusting quantities based on the population size in each site and removing medicines that required an import license.

4. Strategic planning workshop: In the first month of implementation, program staff involved in the management and supervision of programs were invited to a two-day workshop in Juba, including the medical coordinator and/or midwife supervisor from each site. Based on guidance in the *Field Guide*, the workshop led to the development of an action plan that identified specific activities needed to implement the newborn interventions, such as supportive tools and organizational protocols.

### Data collection

Multiple perspectives and data sources were used in this study to provide an in-depth description in chronological order of implementation at the community and facility level from June to November 2016. All participants provided verbal consent, were aged 18 and older, and had completed either the clinical training or planning workshop. Ethical approval was received by the Republic of South Sudan Ministry of Health.

Facility- and community-based health workers were invited to participate in a focus group in each site. Groups discussions were conducted by trained locally-based research assistants to generate a consensus on the quality of the intervention design and how the intervention was implemented at each site. Study co-investigators developed separate semi-structured guides for the community and facility discussions, based on the CFIR framework (see Additional file 1). Guides were translated to Arabic and Nuer languages, and verified by local researchers for accuracy. Seven focus groups were held with health workers in the first month of implementation and 12 focus groups were conducted after 3 months using the same study procedures, representing a total of 61 CHWs and 43 facility-based health workers (Table 1). Discussions comprised six to eight participants, took approximately 90 min, and were audio-recorded and facilitated by a research supervisor with two notetakers. Recordings were translated and transcribed into English by a notetaker, and then reviewed by the facilitator for accuracy.

**Table 1:** Participant characteristics for qualitative data, June – November 2016

Variable	Focus groups (*N* = 19)		In-depth interviews (*N* = 7)	
	No.	(%)	No.	(%)
Sample size				
Number of participants	104		7	
Location				
Juba	21	(20.2)	4	(57.1)
Malakal	32	(30.8)	1	(14.3)
Maban	51	(49.0)	2	(28.6)
Type of staff				
Community health worker	61	(58.7)	0	(0.0)
Facility-based TBA	13	(12.5)	0	(0.0)
Midwife, clinical officer or nurse	30	(28.9)	0	(0.0)
Senior manger	0	(0.0)	7	(100.0)
Residence of staff				
Local	74	(71.2)	0	(0.0)
Non-local	30	(28.8)	7	(100.0)
Sex				
Male	61	(58.7)	5	(71.4)
Female	43	(41.3)	2	(28.6)
Mean age, years	33.1 ± 8.9		36.3 ± 2.4	
Education completed				
None	8	(7.7)	0	(0.0)
Primary	29	(27.9)	0	(0.0)
Secondary or higher	67	(64.4)	7	(100.0)
Mean time employed by IMC, years	1.8 ± 1.4		2.6 ± 1.3	

During the last month of study implementation, a study co-investigator facilitated interviews in English with seven senior managers who were involved in the planning, supervision and management of day-to-day operations during the study period. The interview guide, based on the same framework used in the focus groups, gathered detailed information on implementation activities (see Additional file 2). Interviews were conducted online using Skype (Version 7.52), lasted an average of 66 min, and were audio recorded with the participant’s consent.

Direct observation through health facility assessments was done to document specific aspects of the health system including medical commodities, service delivery and information systems. Research assistants visited study facilities monthly to record availability of supplies, infrastructure, and delivery and newborn admission registers. Electronic, structured checklists on a mobile device were used to document observations. Due to intensified conflict and violence, no facility assessments were conducted in July.

Lastly, we collected a variety of documents, including national policies for reproductive health, humanitarian response plans, copies and photographs of posters and other educational materials, health facility and community registers, facility charts of monthly reporting statistics, supervision checklists, medical supply consumption reports, and staffing plans. These documents were used to provide additional context on the health system building blocks for newborn health service delivery in camp-based settings and to draw linkages with primary collected data.

### Analysis

Data were analyzed in two phases by study co-investigators. The goal of the first phase was to individually analyze each data source; in the second phase, we distilled results into summaries for each health system building block. For the qualitative data, transcripts from focus groups and in-depth interviews were uploaded to NVivo (QSR International, Version 11.4.0) for qualitative coding and thematic analysis. Sections of transcripts were coded into categories and sub-categories to discern patterns emerging from the data. This initial coding was used to develop a codebook along with the CFIR framework and input from local research assistants (Table 2). Then, all transcripts were recoded using the final codebook. A research assistant coded a subset of the transcripts, and analytical memos were compared with those of the primary author for additional insight.

**Table 2:** Summary of emergent themes for qualitative data

Theme	Emergent Sub-Theme
Individual characteristics	*Knowledge or beliefs*: Knowledge of benefit for mothers and/or newborns, or values placed on the importance of the task
Intervention characteristics	*Complexity*: Perceived difficulty of implementation reflected by duration, disruptiveness, or number of steps required to implement
	*Adaptability:* Degree to which an intervention was adapted to meet local needs
	*Design quality:* Perceptions on how the intervention was developed or presented
Inner setting	*Readiness:* Leadership engagement, available resources, or access to information
	*Culture:* Clinical protocols or expected staff roles and responsibilities that guide health workers’ behavior
	*Climate:* Extent to which use of that intervention is supported or expected within the organization
Outer setting	*Patient acceptance:* Factors that influence use or accessibility by community; attitudes of the community towards the intervention
	*Policies and funding:* External strategies that influence interventions including national policies and donor alignment
	*Conflict:* Impact of the conflict on insecurity or shift in services or community needs

Data from the health facility assessments were entered on Android tablets using Magpi mobile data software (DataDyne Group LLC, Version 3.2.2). Data were initially monitored for quality by a field supervisor and then uploaded to an online platform. Study co-investigators subsequently reviewed forms for internal consistency and exported data to Stata (StataCorp LP, Version 13.1) for descriptive analysis. Missing responses were excluded from analysis. For document analysis, a list of compiled materials was indexed in an annotated bibliography in Microsoft Excel describing the source, date, and data. The primary author systematically reviewed the materials for information relevant to the health system building blocks in camp-based settings and summarized data into a spreadsheet.

## Results

The findings are presented in the context of the six WHO Health System Framework building blocks.

### Leadership and governance

In the context of national policies, the Reproductive Health Strategic Plan 2013–2016 from the South Sudan Ministry of Health identifies improving newborn health as a priority. We found that the national strategy addressed broad packages such as skilled care at birth, basic emergency obstetric and newborn care (BEmONC), access to facility-based deliveries, antenatal and postnatal care, and implementing a maternal and neonatal death review system. However, the policy lacked specific actions for addressing the leading causes of neonatal mortality such as care for small babies, resuscitation and treatment of infections. Similar to the national strategy, which sets no target for improving neonatal mortality as it has for other reproductive health indicators, the 2016 Humanitarian Response Plan addresses services for children under five while failing to expand on critical services for newborns. Some participants from the qualitative interviews argued that the lack of policy support inhibited their ability to expand implementation to other sites or plan for activities in the long term. A senior manager described the impact of health policies, “The first thing is to have support at the policy level. The moment we submit any proposal you get acceptance because there is support from the [health] sector, so then you have all the resources ready from the different UN agencies. But, if you mention newborn no one will listen. There is no policy support for that.”

Several senior managers said that implementation of the newborn activities in the community health component in particular was poorly coordinated with field teams. This led to weak integration of these activities into existing maternal health services. A few pointed to the effective uptake of newborn interventions being facilitated by greater access to a focal person for reproductive health. While prior to implementation community health activities focused on mental health, gender-based violence, and nutrition, discussions with CHWs demonstrated their motivation to implement recently learned newborn interventions. However, the failure to prioritize newborn activities among program supervisors was described as a key challenge. Even when a day was specifically allocated for newborn activities in the community health program’s weekly schedule, CHWs were still unable to reach all newborns for a postnatal home visit within the first 48 h after birth because of competing priorities.

Beyond limited national policies and leadership for newborn health in the humanitarian response, the study sites were heavily impacted by an increase in conflict and violence in July 2016, followed by periods of localized insecurity. Findings from the qualitative data indicated that senior managers, including clinical supervisors, could not access facilities and communities, and some were required to leave the country during the acute period. As a senior manager in one site noted, “The security situation is also a challenge, whereby sometimes we are unable to go to the camps and there are no supervisors to oversee deliveries”. Facing a similar issue in another site during the July crisis, a senior manager reported, “To mitigate the challenge, we recruited two national doctors for each camp to oversee activities 24/7”. National health workers, who were based in the camp and held a leadership role, could coordinate response activities with the field team when expatriate supervisors were unable to access sites. Despite efforts to maintain quality of care during the crisis, health workers felt that decision makers did not prioritize infection prevention for newborns when setting up interim spaces for maternity services, despite the fact that infections are a leading cause of neonatal death. A facility health worker commented, “The challenge is the beds were not enough for admitted mothers so postnatal and antenatal mothers were admitted in the same ward and we discharged them soon after delivery because of space”, and another midwife confirmed that the maternity ward was integrated with the general inpatient ward.

### Health financing

Program managers strongly expressed their frustration with donors failing to prioritize newborn-specific activities in humanitarian funding proposals. We found that the Reproductive Health Strategic Plan advocates for donors to integrate activities described in the strategic plan, including a limited number of neonatal services, into funding requests. Some respondents pointed out that newborn health is seen as a ‘new’ activity, beyond what is ‘traditionally known to be accepted’, so it is often excluded from humanitarian response proposals out of fear of losing funds. As one senior manager observed, “donors are ready to listen when you talk about maternal and child health, gender-based violence, and epidemics; there is less understanding when you talk about newborns*”.* It was further explained that within organizations, program managers lack technical expertise on newborn health and, as a result, missed opportunities to include interventions for this population in funding proposals. These bottlenecks were considered critical barriers to implementing interventions for small and sick newborns prior to the study and will continue to be a barrier for sustaining activities in the long term.

### Health workforce

All respondents confirmed that the severe shortage of SBAs was the main workforce bottleneck for implementing quality newborn care. In facilities, staffing mainly consisted of facility-based TBAs in remote settings of Malakal and Maban as of June 2016, while facilities in the capital city of Juba were exclusively operated by trained midwives. Due to the level of skill required, the absence of SBAs prevented health facilities outside of the capital from providing consistent care. Discussions with facility-based health workers also revealed that staff had little time to care for small and sick newborns because of the time taken from providing antenatal and postnatal care, although some also felt they were not adequately trained to provide what was perceived to be advanced care. During the acute period of crisis, in the first month of implementation, half of the maternity staff in one site left their jobs for safety. The effect of turnover on implementation was two-fold: (a) most new hires were recent graduates or community midwives who required additional on-the-job training, and (b) high workload did not allow time for a second round of clinical training until 4 months later. As a result, during each shift new hires were paired with another midwife that had received the newborn training.

In community health programs, a number of CHWs agreed that a wide scope of work and few workers to cover the large populations were factors that limited time spent on newborn care. A CHW explained effects of the reduced number of CHWs on implementation of home visits: “Before, each individual was responsible for 50 households but now you are responsible for three blocks…it is impossible for us”. Workload for CHWs was exacerbated during the July crisis when they had to shift priorities to the cholera outbreak and increased incidents of GBV, as a CHW described: “It is not easy, you will be assigned different areas each day but sometimes you need to see a baby daily…this is not possible since newborn care is new in the system and we have other tasks…sometimes you go and other emergencies come up”.

### Essential medical commodities

Medical commodities for maternal and newborn health services in South Sudan are mostly procured and distributed using the Inter-agency Reproductive Health Kits, according to the Reproductive Health Strategic Plan. From the document review, we found that these kits did not provide 41 out of 51 recommended (i.e. as outlined in the *Field Guide*) newborn supplies for primary health facilities, and provided no supplies at the community level.

All senior managers confirmed that the availability of essential medical commodities for newborn care improved following the study intervention; however, several health workers expressed that access to these supplies was often disrupted because of poor supply management. Health facility assessments, conducted monthly, demonstrated that half of the study facilities maintained stock of the procured newborn supplies over the six-month study period. Yet, some facilities faced challenges in avoiding stock outs. From the facility assessments, we found that up to nine newborn supplies were unavailable in each given month, varying in the type of commodity that was unavailable. Supplies were available in the warehouse (based on stock cards) but likely not monitored in the facility. A senior manager noted that better management of supply chains through weekly tracking could help ensure facilities are always equipped to provide care, and particularly ready in the case of an acute crisis that weakens the health system infrastructure and limits external support.

### Health service delivery

Evidence from our case study data showed that relative to those in Juba, health facilities outside of the central capital faced greater bottlenecks to delivering quality newborn care. These PHCCs relied on facility-based TBAs to provide maternity care, faced frequent power outages, and had occasional disruptions in running water. That being said, even facilities in the capital city encountered 10 days without running water during the initial month of acute crisis in July 2016. With the exception of one site, readiness to deliver basic emergency obstetric care (BEmOC), based on six signal functions, was maintained at the hospital and PHCCs over the study period. In-person observations also found that supplies such as a neonatal-sized bag and mask for resuscitation, weighing scale, fetal heart rate monitor, partograph, and hand soap were readily available in the labour and delivery area. Most health workers were critical of the lack of sufficient space in the maternity ward and argued that this affected newborn care in several ways. Mainly, they were unable to keep essential newborn supplies in the postnatal ward, which prevented admission of newborns with sepsis or low birth weight, and instead cases were referred out of the camp. Other aspects, such as the excessive heat in the maternity ward, were barriers to incentivizing mothers to have facility-based deliveries or stay in the facility for at least 6 h after delivery before being discharged.

Almost all respondents agreed that challenges to delivering quality newborn care were primarily attributed to lack of 24/7 skilled care, as described above. Another feature of skilled care noted by participants was that clinical protocols require a higher-level health worker such as a clinical officer to assess small and sick newborns and approve treatment (e.g. injectable antibiotics for sepsis). Senior management recognized that simpler protocols are needed so that midwives and nurses can take on additional responsibilities for these newborns.

Checklists for supportive supervision, developed as part of the study intervention, were conducted on a weekly basis with community- and facility-based health workers. Use of checklists was delayed for the first 3 months at all sites due to the impact of the July 2016 crisis on supervisor presence and time. Once available, from September to November, we found that the newborn practices that required additional reinforcement were measuring the newborn’s temperature in the first 2 h, ensuring immediate skin-to-skin contact between mother and newborn, neonatal resuscitation, maintaining hygiene standards, and providing health education on newborn danger signs. KMC practices were often forgotten by health workers because of the low number of small babies.

Bottlenecks for postnatal home visits by CHWs, which were described earlier, persisted throughout the implementation period. In addition, CHWs argued that midwives were not documenting a mother’s address before discharge so they could not locate households. Senior managers recognized that the weak delivery of community newborn services was mainly due to low numbers of CHWs per households and the lack of involvement of community health supervisors. Recent clinical training with ongoing supportive supervision, pictorial educational materials, and community acceptance of practices were reported to enable health workers to implement community-based newborn interventions.

With regard to the referral system, participants noted: (a) newborns were often referred to hospitals outside the camps for advanced resuscitation, severe infections, and complications during labor without knowing the quality of services available at referral facilities; (b) emergency transport was often unavailable due to availability of drivers or due to insecurity; and (c) distance and lack of transport to facility within the camps caused mothers to delay or miss postnatal visits. This is how one health worker explained the effect of poor referral systems during acute crisis: “Before the crisis we were sending more referrals to the tertiary hospital [outside of camp], but now there are no referrals due to insecurity along the way. The UN is not allowing people to go out and this is a big challenge especially at nighttime. Many mothers delivered low birth weight and premature babies, but there were no ambulances at night. Even today we lost a baby due to lack of referral for a complicated delivery”. A senior manager described an enabling factor for referrals among CHWs: “We introduced a referral form for CHWs to refer to the facility…it has pictures that show a newborn with a fever, for example. Because we don’t have very many people who are literate, the gateman can look at the picture in the referral form and immediately accept the patient”.

### Health information system

In-person observations found that most study facilities consistently recorded the birth outcome and weight of newborns in their delivery registers during the study period. Yet documentation of newborn information was based on the health workforce. Health workers noted that TBA-staffed facilities had incomplete delivery registers. Furthermore, newborn admissions were not well documented in study facilities. One site introduced a newborn register during implementation, but health workers reported it was too time consuming to complete. Overall, senior managers felt that staff, particularly new hires, do not prioritize documentation. As a result, senior managers sought to improve tracking of newborn activities during the study but faced resistance from their peers because newborn indicators were not required in donor reporting. Several health workers and managers pointed out that CHWs improved documentation of referrals and home visits for newborns during the study, although there was inconsistency in the data captured across sites.

### Community engagement

In the national reproductive health strategy, community mobilization for maternal and newborn health using mass media campaigns and peer educators is a priority action. Two aspects of community engagement were discussed among health workers in focus groups. The first one was the overall positive interaction between caregivers and health workers. Receptive attitudes among mothers allowed facility- and community-based health workers to deliver health messaging on newborn care. However, CHWs admittedly preferred that materials be translated to the local language and be more accessible to households using mass media. Another feature was the resistance some health workers faced from the community about traditional harmful beliefs and opposition to ‘western medicine’, which they noted requires additional health education by CHWs. A facility health worker explained, “when we send a mother back in the community, the grandmothers believe in applying something on the cord and then they come back with sepsis and other things”. Relationships between caregivers and CHWs were generally positive during home visits, except caregivers were uncomfortable with CHWs handling newborns. A CHW noted: “When you make a postnatal visit sometimes the caregivers don’t allow you to see the baby, and they see the baby as delicate so they don’t allow others to touch newborns”. Several CHWs felt that engaging community leaders, husbands, and the elderly could address these bottlenecks and encourage better newborn practices.

## Discussion

Reducing stillbirths and neonatal mortality in conflict-affected countries requires addressing health system bottlenecks. This study contributes to the limited body of evidence on maternal and newborn care during conflict, using the WHO Health Systems framework adapted by the *Every Newborn Action Plan* [21]*.* Our findings present the main bottlenecks that influenced the implementation of newborn interventions by an international humanitarian organization in communities displaced by ongoing conflict in South Sudan. The findings confirmed that there are critical bottlenecks that are applicable across many humanitarian responses, such as weak national policies, limited resource allocation, poor supply management, and destroyed infrastructure. However, barriers to implementing newborn care were more nuanced when we analyzed intervention-specific activities. We identified three main health system building blocks for community- and facility-based newborn programming among displaced communities in South Sudan: leadership and governance for comprehensive neonatal services, health workforce for skilled care, and service delivery for care of small and sick newborns. These factors alongside potential solutions identified through the qualitative interviews and existing literature are described in more detail below (Table 3).

**Table 3:** Summary of bottlenecks and solution themes

Health system building block	Subcategory	Common bottlenecks	Solution themes
Leadership & governance	Policy and planning	Absence of strategies to address premature babies and neonatal sepsis	• Review and improve existing policies, and sensitize health officials and donors on newborn care
	Supervision	Non-local staff had restricted access to sites during acute conflict or evening hours	• Increase hiring of local staff who can provide continuous newborn care
Health financing	Policy	Newborn-specific interventions are outside of humanitarian response	• Advocate for donors to include newborn care in humanitarian funding opportunities • Increase knowledge among program managers to integrate newborn interventions in maternity programs
Health workforce	Skilled care	Shortage of skilled health workers to continuously provide comprehensive package of interventions	• Authorize task-shifting where appropriate and invest in training institutions
	Training	Lack of training for recent graduates from national institutions on newborn care	• Integrate comprehensive curricula on newborn care into national institutions
	Number and distribution of CHWs	Limited coverage and wide scope of work to conduct home visits in first 48 h after birth	• Increase number of CHWs to meet Sphere standards or dedicate cadre for maternal and newborn activities by transitioning TBAs to CHWs
Essential medical commodities	Integration	Lack of newborn supplies in Inter-agency Reproductive Health Kits	• Coordinate with UN agencies to integrate newborn commodities into existing supply chains
	Supply chain management	Stock out of supplies in facilities over time	• Improve tracking of supplies and improve forecast of newborn supply needs to buffer for times of acute crisis
Health service delivery	Infrastructure	Limited space for neonatal admissions	• Prioritize standards to allocate hygienic areas for newborn care
	Clinical protocols	Unavailability of higher-level health workers to care for small and sick newborns	• Adopt simpler protocols for mid-level cadre to take on additional responsibilities
	Referral system	Poor quality of care for small and sick newborns at referral facilities Lack of access to referral facilities (transport and distance)	• Strengthen newborn care at primary care level • Partner with security personnel (e.g. UN peacekeepers) to plan for referrals during insecurity
Health information system	Quality	Incomplete registers and limited data on neonatal admissions	• Integrate critical newborn health indicators in donor reporting • Introduce pictorial registers for illiterate health workers
Community engagement	Personal beliefs	Barriers to handling newborns by CHWs	• Increase awareness and engagement of community leaders

First, humanitarian response plans and national policies are often the basis for decision-making when donors fund proposals. Excluding key neonatal interventions and packages in these strategic plans can prevent organizations from seeking or receiving funding to address the top causes of neonatal death. Fortunately, tireless advocacy has increased funding for reproductive health issues in humanitarian emergencies. A recent analysis reported that the number of funded proposals for humanitarian assistance that included reproductive health increased by an average of 21.9% per year from 2002 to 2013, which mostly supported maternal and newborn health activities [30]. Despite this, neonatal-specific interventions such as resuscitation, care for small babies and treatment of infections were not considered part of the humanitarian response in South Sudan, leading to missed opportunities for integrating care for small and sick newborns in a country mainly surviving off humanitarian assistance [31]. Similarly, bottleneck analyses conducted by the *Every Newborn Action Plan* in non-conflict affected settings found that failure to integrate newborn activities, such as KMC or inpatient care, in national policies or budgets can significantly impede financing of these activities [32]. Greater advocacy among government and donors to invest in more comprehensive packages of essential interventions, which address the main causes of maternal and neonatal mortality, could prevent organizations from choosing between life-saving services [33].

Second, skilled care at birth is recognized as one of the most lifesaving interventions in maternal and newborn health, reducing neonatal mortality at birth by 25–40% [5]. However, the availability of a skilled workforce is the greatest bottleneck for achieving skilled care at birth in the majority of low-income countries [34]. South Sudan’s national strategy for reproductive health has clearly prioritized building the cadre of front line health workers such as midwives and nurses through their training institutions. It is vital for pre-service education to better address newborn care to avoid the need for time-intensive on-site training, which was shown to be unmanageable during acute crisis. Building the health workforce in South Sudan is an undeniably daunting task when continuous insecurity causes health care workers to flee for their lives to nearby countries [35]. Task shifting, a frequent solution in countries facing a shortage of health personnel, has been accepted in South Sudan as a way to increase access to emergency care through mid-level health workers [36, 37]. Facility-based lay health workers like TBAs can be trained, within WHO recommendations, with regular supportive supervision and coaching to promote newborn care practices and KMC for low birth weight babies. With additional training, TBAs can also improve access to treatment for neonatal sepsis by administering antibiotics [38]. Although severe infection, extreme preterm, and very low birth weight require specialized care to prevent mortality, less experienced staff can support the management of these newborns with the right equipment and supervision [32, 39]. In the absence of supervision, as seen during the acute phase of the July crisis, contingency plans should be in place to strengthen referral pathways with support from security personnel. Findings from our study demonstrate that CHWs are also an important resource in the community for timely identification of neonatal danger signs, which has also proven to be effective in increasing treatment for neonates in Nepal [39, 40]. Although evidence shows that home visits early in the first week of life can significantly reduce neonatal mortality, the implementation of this activity was proven difficult in our study sites where CHWs have a very wide scope of work [41–43]. Increasing the number of CHWs to meet Sphere standards and strategically planning their activities may improve their ability to reach newborns in the first 48 h after delivery or discharge [44, 45].

Third, delivery of health services in displaced person camps allows for greater access to facility-based care and regular follow-up through CHWs [10]. However, the sudden onset of violence and instability in July introduced additional bottlenecks, such as disruptions in referral systems and follow-up care, in the relatively well functioning health systems within the camps. Health workers were concerned about poor infection control and limited space for newborn admissions when maternity services were combined with the in-patient ward. These moments can be especially life threatening for sick or premature babies [32]. As the complexity of providing care increases during acute crisis, outpatient care and recurrent home visits could improve management of premature, yet stable newborns [32]. Standard precautions and supervision should ultimately be prioritized when setting up health services for vulnerable populations such as pregnant women and newborns. Lastly, community engagement is recognized as a critical component of implementing community-based maternal and newborn services [46]. In our study, caregivers were initially resistant to CHWs handling newborns while assessing their danger signs, requiring additional health education, engagement of community leaders and endorsement from the formal health system.

Our findings demonstrate the effect of acute crisis on the implementation of community- and facility-based newborn care, and may be relevant for other conflict-affected settings. We also found that some of the bottlenecks are similar to those in low-income countries with limited resources. Health workforce and service delivery were key bottlenecks in other low resource settings not affected by conflict [21]. However, the acute conflict and the resulting insecurity as well as the role of humanitarian actors in running and operating health facilities, made the nature of the bottlenecks, and the potential solutions, different from other country contexts. This study’s analysis also generated several programmatic and policy solutions that can contribute to improving guidance in humanitarian crisis. Further research to improve the implementation of community and facility level newborn interventions in settings with ongoing conflict is crucial. Understanding the feasibility of guidelines recommended in contexts such as South Sudan would allow for context-specific adaptations and innovations.

### Limitations

Several limitations should be considered when assessing our findings. First, our case study is contextualized to the selected time period (June to November 2016) and camp-based settings of South Sudan. Our findings are likely less transferable to conflict-affected countries in middle-income countries with stronger health systems. The credibility and dependability of our study, which can be affected by social desirability bias, was addressed by providing rich data from multiple data sources and perspectives; using observations and secondary documents confirmed perceptions found in qualitative data. However, we did not assess the building blocks framework with the same depth or number of stakeholders as was done in the Every Newborn bottleneck analyses. Lastly, the July crisis impacted our ability to collect primary data in the second month of implementation. We attempted to address this gap in data by exploring the impact of the crisis in the next month’s data collection activities.

## Conclusions

Reaching the Sustainable Development Goals by 2030 requires a cessation of conflict in South Sudan, as well as considerable investment in the nation’s health system. Newborn survival in conflict-affected settings depends on identifying local solutions to address the bottlenecks in implementing essential community and facility-based interventions that can withstand the destabilizing impact of conflict, migration, and other factors. It is critical to devote resources for emergency preparedness and response toward improving access to skilled care at birth and newborn medical commodities, advocating for more comprehensive national policies, and strengthening referral systems. Newborn care should be a priority in health activities at the onset of an emergency, and no longer excluded from humanitarian response and funding commitments. International organizations and donors supporting health programs in these contexts, which are largely reliant on international aid, play an integral part in reducing deaths among the most vulnerable group in displaced populations.

## Additional files

Additional file 1: Focus group discussion guide. (DOCX 114 kb)

Additional file 2: In-depth interview guide. (DOCX 113 kb)

## Abbreviations

ANC: Antenatal care
CHW: Community health worker
GBV: Gender-based violence
IDP: Internally displaced person
IMC: International Medical Corps
KMC: Kangaroo mother care
PHCC: Primary health care center
SBA: Skilled birth attendant
TBA: Traditional birth attendant
WHO: World Health Organization
