# Supplementary material for: Understanding health systems to improve community and facility level newborn care among displaced populations in South Sudan: a mixed methods case study
Source: BMC Pregnancy Childbirth. 2018 Aug 10;18:325. doi: 10.1186/s12884-018-1953-4 (PMC6086013; doi:10.1186/s12884-018-1953-4)
Supplement: Supplementary file 1 — Focus group discussion guide. (DOCX 114 kb) [file 12884_2018_1953_MOESM1_ESM.docx]

FOCUS GROUP DISCUSSION GUIDE

(Facility Health Workers)

**Feasibility of Implementing the *Newborn Health in Humanitarian Settings Field Guide* to Improve Newborn Care Practices in South Sudan**

1. **FGD details.**

Data Collection Date _______________________ # of Participants _______________________

Start time _______________________ End Time _______________________

Site _______________________ FGD Location _______________________ Audio File # _______________________

Facilitator _______________________ Notetaker _______________________

Transcriber _______________________ Transcription Date _______________________

1. **Complete oral consent.**
2. **Welcome and ground rules.**

Welcome to our group discussion. My name is ______________ and I will be facilitating today’s session. Joining me today are ­­­­­­­­­­___________ who will be taking notes and ____________ will be here to assist me.

Here are a few ground rules before we start:

- First, please turn all cell phones to silent.
- Please speak clearly so that our tape recorder can pick up your voice.
- Please speak one at a time.
- Please do not have side conversations
- Please give each other a chance to speak
- There are no right or wrong answers, and we will have different points of view. We encourage you to talk to each other, to add thoughts to others’ comments, and to share reactions or disagreements with respect.
- And we ask that you please respect the privacy of everyone here and do not share the content of this discussion outside this room.

**Introduce the topic:**

- Please remember the IMC newborn training for health workers that was completed two months ago to train health staff on how to provide care for newborns.
- The training included fetal monitoring, using the partograph, immediate newborn care, resuscitation, breastfeeding, managing infection, and danger signs for the baby.
- The training used videos, role plays, demonstration, and hands on practice with the mannequins.
- Now I would like to ask you some questions about the training.

1. **FGD Questions**

Topic 1: Newborn Care Services

1. It has been about two months since you attended the IMC newborn training. What comes to mind when you remember this training? Do you remember the information and how you felt at the time?
2. How did the training help you understand what information is important to share with pregnant women and women who just delivered a baby? Is there any information missing that would be helpful to know?
3. What advice do you have to improve the IMC newborn training? [**Probe**: What type of information? More/less? How the information was presented?
4. Now let's talk about the topics that you learned in the training. In the past two months, have you had experiences with providing health care for women in labor and delivery? What care did you provide? [**Probe**: Use of partogram and fetal heartbeat monitoring]

- Now I want you to think back to these experiences. What were some of the biggest challenges in providing health care for women in labor and delivery? [**Probe**: How difficult or comfortable were you with the task? Did it take away time from your other tasks? How much of your time did this take? Was making decisions on your own a challenge?]
- How does the community influence how you care for pregnant women? [**Probe**: What are the challenges? How did they support what you say? Do most women deliver at home or the facility?]
- How did you handle those challenges? What do you feel might help make those challenges less challenging?

1. In the past two months, have you had experiences with providing postnatal care for healthy babies within the first 24 hours after delivery? What care did you provide? [**Probe**: keeping the baby warm? Breastfeeding support? Cord, skin or eye care? Counseling on danger signs? Providing Vitamin K? Providing vaccinations? Weighing the baby? Giving a birth certificate?]

- Now I want you to think back to these experiences. What were some of the biggest challenges in providing postnatal care for healthy babies? [**Probe**: How difficult or comfortable were you with the task? Did it take away time from your other tasks? How much of your time did this take? Was making decisions on your own a challenge?]
- How does the community influence how you provide postnatal care? [**Probe**: What are the challenges? How did they support?]
- How did you handle those challenges? What do you feel might help make those challenges less challenging?

1. In the past two months, have you had experiences with providing health care for a small or sick newborn? What kind of sickness? What care did you provide? [**Probe**: Newborn resuscitation with stimulation or bag and mask? Keeping the baby warm through KMC? Infection-prevention? Breastfeeding support? Monitoring of vital signs? Antibiotics for newborns for sepsis? Immediate treatment and referral of newborn with signs of sepsis to hospital?]

- Now I want you to think back to these experiences. What were some of the biggest challenges in providing care for a small or sick newborns? [**Probe**: How difficult or comfortable were you with the task? Did it take away time from your other tasks? How much of your time did this take? Was making decisions on your own a challenge? How was the referral process?]
- How does the community influence how you care for small or sick newborns? [**Probe**: What are the challenges? How did they support?]
- How did you handle those challenges? What do you feel might help make those challenges less challenging?

1. How does communication among health workers effect how you care for newborns?
2. How often are you supervised? Does your supervisor provide you with feedback? How does the supervision impact how you care for newborns?
3. How did the crisis impact your work? [**Probe**: How did it affect deliveries? Postnatal care? Care for small or sick babies? Supervision?]
4. Is there anything that can be done to prepare you better for providing this newborn care? [**Probe**: Training? Supplies/Medicine? More time to follow up on activities?]
5. Overall, did you feel that you were able to implement all the newborn health practices that were recommended in the training? Was there anything that prevented you from providing all recommended services?

Topic 2: Communication Materials

1. During the training, we used these communication materials to educate pregnant women and mothers on newborn care and danger signs. How did women and their families react to these materials? [**Probe**: Did they understand the materials?] [If not used, ask why materials have not yet been used.]
2. What suggestions could you give IMC to make these communication materials more clear to pregnant women and mothers? Would you use these same materials, or would you do it another way?

Topic 3: Newborn Registers

1. Now I want to ask you a few questions about recording information about newborns. What is your experience in using the registers to record information about newborn admissions? What might make someone fail to use the register or complete the register? [**Probe:** Are you comfortable with using it? Does it take away time from your other tasks? How much of your time does this take? ]
2. What suggestions could you give us to make registers easier to collect information on newborn admissions? Would you use the existing registers, or would you do it another way? Is there information that the registers do not collect that they should?

Topic 4: Closing Group Discussion

*[Explain that drinks and snacks will be available at the end of the discussion. Summarize the main points from the group discussion and ask for consensus on main points.]*

1. Before we end our discussion today, does anyone have any additional thoughts that were not shared?

Thank you again for your help and for sharing your opinions with us today. We really appreciate your time and contribution. Feel free to ask any questions.

1. **FOR SUPERVISOR: Complete FGD Participant Profile**.

| **Participant Number** | **Training** | **Age**  **(Years)** | **Gender** | **Number of months with IMC** | **Type of health worker** | **Highest Education Level Achieved** |
| --- | --- | --- | --- | --- | --- | --- |
| Codes | 0 = No  1 = Yes |  | 1 = F  0 = M | 0 = less than one month | 1= Midwife  2 = Midwife Assistant  3= Nurse  4= Nurse Assistant  5= Doctor  6= Clinical Officer  7= CHW  8= RH promoter  9= TBA | 0 = None  1 = Primary  2 = Secondary or higher |
| **1** |  |  |  |  |  |  |
| **2** |  |  |  |  |  |  |
| **3** |  |  |  |  |  |  |
| **4** |  |  |  |  |  |  |
| **5** |  |  |  |  |  |  |
| **6** |  |  |  |  |  |  |
| **7** |  |  |  |  |  |  |
| **8** |  |  |  |  |  |  |
| **9** |  |  |  |  |  |  |
| **10** |  |  |  |  |  |  |

Notes:
