# Supplementary material for: Understanding health systems to improve community and facility level newborn care among displaced populations in South Sudan: a mixed methods case study
Source: BMC Pregnancy Childbirth. 2018 Aug 10;18:325. doi: 10.1186/s12884-018-1953-4 (PMC6086013; doi:10.1186/s12884-018-1953-4)
Supplement: Supplementary file 2 — In-depth interview guide. (DOCX 113 kb) [file 12884_2018_1953_MOESM2_ESM.docx]

IN-DEPTH INTERVIEW GUIDE

(Program Staff)

**Feasibility of Implementing the *Newborn Health in Humanitarian Settings Field Guide* to Improve Newborn Care Practices in South Sudan**

Data Collection Date Site/Facility

Start time/End time ________________________ IDI Location

Participant # ________________________ Audio File #

Data Collector Notetaker

Transcriber ________________________ Transcription Date ____________________

Participated in program staff training **Yes No**

Thank you for meeting with me today. The questions I am going to ask do not have right or wrong answers. We are interested in learning about your experiences with implementing activities that are related to the provision of newborn health services.

**PLEASE START HERE ANSWERING QUESTIONS HERE:**

Topic 1: Introduction

Gender: Male ____ Female

1. Can you tell me your age at your last birthday? ______
2. How long have you been working in South Sudan?
3. How long have you been working with IMC?
4. What is your current position with IMC? Could you tell me how long you have been working in this position?
5. Can you tell me about the roles and responsibilities of this position with IMC? How has it changed since you participated in the newborn training?
6. What are the biggest barriers/challenges you face in your day-to-day work?
7. What were your impressions about the newborn training in June? What did you like the most? The least? How did the information from the training help you? Is there any information missing that would be helpful to know?
8. For the Newborn Field Guide document that was shared during the training-- Do you have any suggestions for changes to make the document more useful?

Topic 2: Development of communication materials

1. Do you have any knowledge of the facility or community health workers sharing information, education or communication (IEC) materials on newborn care with the affected population? [*If no, skip to next topic.*]
2. Thinking about your experiences with the newborn care educational materials-- Can you describe how the IEC materials for newborn care have helped? What made the experience successful?
3. What are some of the biggest challenges in using the IEC materials for newborn care?
4. How did you handle those challenges? Or what do you feel might help make those challenges less challenging?

Topic 3: Development of referral systems

Have you had experience with referring pregnant women or newborns for more advanced care including protocols, procedures and practical guidance to facilitate the transfer? [*If no, skip to next topic.*]

1. Do you have knowledge of the referral system for pregnant women or newborns in your sites? [*If no, skip to next topic.*]
2. What is the protocol for transferring newborns at the community, PHCC or hospital level? How are providers trained on the referral protocols? About how many health facilities currently have a written and functional protocol in place?
3. Can you describe the referral system for newborns that need more advanced care from the community level? PHCU/PHCC? Hospital? [Where do you send them? What is the process for referral?]
4. What information is recorded for outgoing referrals? How do you know if the referral was completed?
5. Thinking about your experiences with the referring newborns, tell me about a success that you or a colleague had. What made that experience successful?
6. What are some of the biggest challenges in the referral system?
7. How did you handle those challenges? What do you feel might help make those challenges less challenging?

Topic 4: Procurement and Distribution of Newborn Care Supply Kits

1. Do you have experience with distributing or using the newborn supply kits that were sent in June? [*If no, skip to next topic.*]
2. Thinking about your experiences with the newborn supply kits, tell me about how the newborn supplies have been helpful for providing care.
3. What are some of the biggest challenges in using the newborn supplies?
4. Have you heard of other medicine, supplies or equipment that are useful for newborn care but are not included in the kit?
5. Have you heard of medicine, supplies or equipment that are included in the newborn supply kit but were not used or needed?

Topic 5: Implementation of M&E Plan

1. Do you have experience with monitoring newborn health data (e.g. birth/death registers, newborn admissions)? [*If no, skip to next topic.*]
2. What are some of the biggest challenges in collecting or reporting newborn health data?
3. How do health facilities follow up on maternal or newborn deaths? Near misses? About how many health facilities currently conduct a review of maternal deaths? Newborn deaths? Near misses?
4. Does the data differentiate between stillbirths and neonatal deaths after birth?
5. Are complications that occur in the first 28 days of life recorded?

Topic 6: Provision of Newborn Health Services

1. Have you had experience with providing newborn health care?
2. Have you used the materials in the Newborn Field Guide document to help you provide newborn health services?
3. Thinking about your experiences with the provision of newborn health services, tell me about a success that you or a colleague had. What made that experience successful?
4. What were some of the biggest challenges in providing newborn health services?
5. We understand there is a challenge with having skilled birth attendants present or on call at health facilities due to insecurity. How does the lack of a skilled birth attendant at certain hours impact how you provide care for newborns?
6. Is there anything that can be done to prepare you better for providing newborn health services?
